# New York Academy of Medicine—Section on Orthopedic Surgery

**Published:** 1892-07

**Authors:** 


### Society Proceedings.

### NEW YORK ACADEMY OF MEDICINE.—SECTION ON ORTHOPEDIC SURGERY.

*Stated Meeting, May 20, 1892.*

Henry Ling Taylor, M. D., Chairman.

### CONGENITAL DISLOCATION OF BOTH PATELLÆ.

Dr. S. Ketch presented a little girl who at first glance seemed to have only knock-knee, but on flexing the limbs, a complete dislocation of the patella downwards and forwards was observed, and the dislocation could be readily reduced by extending the limb. The deformity was much more marked on the right side. The condition was probably congenital, although it had not been noticed by the mother until recently, as the child was able to walk with no more difficulty than is observed in an ordinary case of knock-knee. Dr. Shaffer had suggested that this was the opposite of the condition which he had described under the head of elongation of the ligamentum patellæ at the last meeting of the American Orthopedic Association.

Dr. John Ridlon said that he had seen three such cases in the practice of the late Mr. Thomas. The treatment had consisted in hammering the deficient condyle with an egg-shaped wooden mallet, and in two of the cases, the treatment had already effected sufficient development to prevent dislocation, and in the other case the treatment had only been just begun.

Dr. W. R. Townsend said that he had presented some time ago, to the Surgical Section of the Academy of Medicine, a colored girl who could, by muscular action, produce at will a complete dislocation of both patellæ, either to the outer or inner side. A knee-cap was applied, and an effort made to restrict the movements of the fibers of the vastus externus and internus which seemed to be abnormally developed. She was kept under observation for six or eight months, and at the end of this time she could not produce the dislocation at will, and the dislocation occurred quite infrequently.

Dr. N. M. Shaffer said that in his case of elongated ligamentum patellæ, the man had had a fall, which was followed by an outward dislocation of the patella, on the right side. After consultation with several other surgeons, in view of the fact that the intercondyloid notch was filled by an exostosis, it was considered best to make no attempt at reduction, and at present, although the patella lies on the outer aspect to the joint, the man is perfectly able to walk ten or fifteen miles a day. In the case just presented, he did not think the external condyle was deficient, but the ligamentum patellæ was so short that the patella, instead of passing over the trochlea, is drawn down to a point where, owing to the knock-knee, it is very easily dislocated. On this account, he thought that treatment directed towards securing an elongation of the ligament would be more apt to prove successful, than simply hammering the outer condyle.

Dr. Ketch, in closing the discussion, said that he agreed with the last speaker as to the inadvisability of resorting to operative measures. Not long ago he had seen a young lady with a somewhat similar condition. Twelve years before, the patella had been dislocated by muscular action, and this had again occurred shortly before he saw her. Reduction was easily effected by extending the limb.

Dr. Royal Whitman presented several patients :

Case I. A girl, fourteen years of age, to illustrate the appearances in non-deforming club-foot. As in many similar cases, the history was one of awkwardness in walking for many years, with increased pain and discomfort during the past six months. She presented the calluses on the balls of the feet, the contraction and tenderness of the plantar fascia, and the limitation of dorsal flexion to which Dr. Shaffer had called attention in his original communication.

Case II. A woman, fifty-seven years of age, who had suffered from chronic rheumatism for many years. On the left side the contraction of the plantar fascia had thrown the foot into a position of equino-varus. There was no deformity of the right foot, but on both sides a well-marked limitation of the dorsal flexion. The electrical reactions were normal. The condition was similar to non-deforming club-foot, and was apparently the result of a rheumatic inflammation.

Case III. A girl of fourteen, with marked spasmodic flat-foot. The case was presented to illustrate the extreme and progressive deformity and disability in this class of cases, which could be easily and quickly relieved by the method of treatment to which he had on several occasions called the attention of the Society.

Case IV. A girl of eighteen, with persistent abduction of the right foot. Although there was no evident deformity, the foot was held in an abducted position by spasm of the peroneii and extensor longus digitorum muscles. The condition was the result of a slight sprain three months before. The symptoms were, pain, fatigue and insecurity, in walking. The case illustrated the condition in so-called chronic sprain of the ankle, which practically never recovered, because the foot being unbalanced by irregular muscular action, was constantly subjected to injury. When the condition was recognized, a cure could easily be accomplished by restoring the normal muscular action. The patient being etherized, the foot should be forced into a position of extreme equino-varus. All adhesions were thus broken up, and the contracted muscles were stretched. The foot was then placed in plaster-of-Paris, and later, by massage, exercises, and a temporary support, the patient could be completely and permanently cured.

Dr. R. H. Sayre thought that in the fourth case there might have been a fracture of the lower part of the fibula, complicating the sprain, which had been overlooked in the treatment of the case immediately after the injury.

Dr. Ketch thought that the fourth case gave evidence of a possible osteitis about the ankle joint, and this condition should be carefully excluded before adopting the treatment proposed.

Dr. Whitman said that he found no indications of an osteitis in the fourth case, and that there was no history or present indication of fracture complicating the original sprain.

Dr. Shaffer said of the second case, that the exaggerated extension of the toes, and the shorter plantar tissues, were characteristic of non-deforming club-foot. He had seen several cases where the symptoms had not become prominent until the age of thirty-five or forty years was reached, and then, whether there was a rheumatic diathesis or not, all the symptoms would be greatly exaggerated. Many cases showed much less deformity than that exhibited in Dr. Whitman's case. The typical non-deforming club-foot showed little or no deformity as such, unless it was sought for and found in the shortened plantar and post-tibial tissues. The lack of proper length prevents normal antero-posterior movement at the ankle and in the tarsal joints, and the entire weight of the body falls upon the "ball of the foot" in locomotion. It is far more common than is generally supposed, and with the use of the antero-posterior traction shoe, there is no necessity whatever for a division of the resisting tissues.

Dr. R. H. Sayre thought the second case presented very much the condition found in ordinary cases of chronic gout and rheumatism, and he had noticed that after the foot had been manipulated somewhat, she was able to move it much better than before, and could voluntarily flex the ankle beyond a right angle, so that it did not seem to be a case of non-deforming club-foot.

### ANCHYLOSIS OF THE HIP.

Dr. Irving S. Haynes, present by invitation, exhibited a specimen of this condition, which he had found in the dissecting-room of the University Medical College. The subject was a man about twenty-five or thirty years of age. The limb was slightly flexed, adducted, and rotated inwards. A sinus opened about half an inch below Poupart's ligament, and one inch internal to the anterior superior spinous process. It passed backwards, soon divided into two tracts, one leading down to the front of the great trochanter, the other up under Poupart's ligament into the iliacus, and then into the obturator internus muscles; then around the middle of the outer border of the obturator foramen into the cotyloid notch, and so into the hip-joint. The iliacus and obturator muscles, as well as all the muscles acting upon the hip-joint, had undergone extensive absorption and fibrous degeneration. The center of the disease, and the starting point, seemed to have been in the head of the femur, but there was also a focus in the epiphyseal line of the great trochanter, which communicated with that found in the head of the femur by a sinus running through the neck and also opened in front through one or two small openings. Another sinus seemed to have led from the acetabulum through the cancelous portion of the ilium into the iliac fossa, where the opening was surrounded by bony formations. Between the ilium and sacrum there was slight mobility of a gliding nature, which the speaker had never observed before, and which was probably intended to partially compensate for the lack of motion at the hip. There was no evidence of the disease in the capsule of the joint. The abscess cavities were limited to the absorbed portions of the iliacus and obturator internus muscles.

### ARTHRITIS DEFORMANS.

Dr. Haynes also exhibited a specimen of this condition, showing erosion and reproduction of bone, with a depression in the acetabulum, and a disappearance of the ligamentum teres. The motions of the joint were slightly limited in every direction. The specimen was removed from an old subject.

### THE TREATMENT OF LARGE ABSCESSES IN POTT'S DISEASE.

Dr. W. O. Plimpton presented several cases of Pott's disease with large abscesses as an illustration of the treatment which he advocated. He did not favor aspiration, because he thought after this had been done, the abscesses were likely to continue to enlarge and burrow into the tissues. While admitting that abscesses were not infrequently absorbed, he wished to depreciate the let-alone treatment of large abscesses which tend to burrow deeply into the tissues, threatening to inoculate these tissues, often causing mechanical deformities of other parts.

Case I. A boy about twelve years of age, who had had Pott's disease since he was three years old. The disease followed closely upon a blow from a brick. When he first came under the speaker's care last July, he was very anemic and weak, with an afternoon rise of temperature. There was a very large abscess situated beneath the glutei muscles, and there was much deformity of the leg, viz.: apparent shortening, inward rotation, and adduction—caused by the abscess. Free incision evacuated a large quantity of fluid, together with broken-down tissue. An examination with the finger showed no involvement of the joint. The diseased parts were thoroughly curetted with a Volkmann spoon, a counter-opening made, and three drainage-tubes inserted. After washing out the cavity with a weak bichloride solution, the superficial cavity was obliterated as far as possible by means of sutures, and primary union occurred except at the site of the drainage tubes. Two of the tubes were gradually withdrawn. The third one, in front, still remains in for drainage, although it has considerably shortened. The apparent inequality in the length of the limbs has disappeared since the operation, and with a plaster-jacket to support the spine, he is able to go to school, and to play with other children. The discharge is steadily becoming less.

Case II. A girl, seven years old, whose disease dates back to a fall about three years ago. When first seen one year ago last January, there was a moderately large abscess, which was opened, and a tube six or eight inches long inserted. The tube has been gradually shortened until it is now three inches long ; the discharge is diminishing, and the patient's general health has markedly improved. Another case was treated in a similar manner, and has steadily improved since the operation. In all, there had been a gradual reduction of the temperature after the operation. The same precautions were observed as in any cutting operation where it is the intention of the surgeon to secure primary union, and after the operation care was taken to keep the wound and dressing aseptic.

Dr. W. R. Townsend said that the location of the tube in the first case reminded him of an accident which occurred about one year ago. He was hastily summoned to the hospital on account of one of the patients having a hemorrhage. He found that a case of large psoas abscess which had been opened and a drainage-tube inserted three weeks before, had suddenly begun to bleed profusely. The hemorrhage was arterial, and with the assistance of Dr. W. T. Bull, he cut down and found that the pressure of the drainage-tube had caused a large perforation in the femoral artery. He accordingly tied the artery above and below the perforation, and the child recovered without further accident.

Dr. Ketch thought the cases presented very much the appearance of those he had seen in the hospital, when it was the rule to open all abscesses as soon as the abscess approached the surface. They did not seem to him to differ materially in their course from those where the abscesses were allowed to open spontaneously, and he could not see that anything had been gained by this method of treatment.

Dr. Ridlon asked if the drainage-tube had been left in for so long a time for fear that the opening would close up, and necessitate another operation. He had always thought that it was not necessary to leave in the tube more than a few days.

Dr. A. M. Phelps thought that the second case had had a decided advantage over the first in being subjected to the operation at a much earlier stage. The slightest increase in an abscess, in his opinion, warranted prompt incision. He spoke emphatically, because the section had almost been committed to the idea that it was better for these abscesses to take care of themselves. But it must not be forgotten that these abscesses were originally collections of tuberculous material, and that when they became infected with pyogenic germs, as almost inevitably occurs, there will be a rapid burrowing of the pus. Another reason for opening these abscesses is that they exert an injurious effect by the internal pressure of the exudate upon the carious foci in the diseased vertebræ, keeping them bathed constantly, and furnishing a fertile source of the subsequent breaking down of these vertebræ, and of a consequent increase in the deformity.

Dr. Ketch thought that the previous speaker had not correctly stated the position of the section on this subject. He thought it would be more correct to say that they took the ground that so many of these abscesses disappeared spontaneously under proper mechanical treatment, that something more than mere accident was necessary to explain it, and that these collections of pus cause injurious pressure had not been proven. The proof of this would be found in a marked increase in the size of the deformity, but in disease of the dorso-lumbar spine, where these abscesses were the most frequent, this did not occur, and Dr. Myers had recently presented a boy who had had two large iliac abscesses disappear spontaneously, and yet there had been no increase in the kyphos as shown by repeated and careful tracings.

Dr. Shaffer said that extensive observation had taught him that with efficient mechanical treatment, the abscesses of Pott's disease almost uniformly pursue a benign course, and he believed that the time would come when those who now operate will see their error. He had seen in the practice of some of the best surgeons in this city, deaths occur after operating upon just such abscesses. When an abscess is very tense, and there are severe local or constitutional symptoms, all recognize the propriety of incision, but, ordinarily, these abscesses are flaccid and do not cause any such "damming up" and injurious pressure as had been described by Dr. Phelps.

Dr. Whitman could see no good reason for waiting until the abscesses appeared below Poupart's ligament. When first discovered, they should be aspirated, and if this fails, iodoform emulsion should be injected. Surely a method of treating the abscesses of Pott's disease, which yielded in the hands of Bruns fifty successful cases out of fifty-two, and of Fränkel, eighteen out of twenty, was one which deserved a fair trial before resorting to severer measures. If aspiration and the injection of iodoform emulsion proved unsuccessful, the method of evacuation recommended by Barker and Treves with immediate closure of the wound, might be employed before resorting to open drainage.

Dr. Plimpton, in closing the discussion, said that the tube had been left in for free drainage, as it had been found that where it was removed shortly after operation, the exuberant granulations choked up the sinus, and gave rise to a great deal more trouble and discomfort than where the tube was retained. At the time of the operation, he had had in mind the possibility of accident from having the tube in too close proximity to the femoral artery, and in this particular case, there were dense cicatricial barriers between the tube and the artery. Small, and not readily accessible abscesses should not be interfered with, unless they cause some disturbance, but he would not hesitate, if circumstances seemed to demand it, to open them above Poupart's ligament. The existence of intra-abscess pressure, and its effect upon the general health, was well demonstrated in one case in which he removed about half a pint of the contents of the abscess by aspiration, with the result of causing an immediate return of the child's appetite, and a prompt relief of his pain. He had seen the iodoform emulsion used in a number of instances without apparent benefit. In considering the percentage of abscesses which disappear spontaneously, it must be remembered that many of them are small abscesses, or are nothing but fluid in the joint, so that the statistics on this point are very defective.

### A CONTRIBUTION TO THE STUDY OF NON-DEFORMING CLUB-FOOT.

Dr. L. W. Hubbard read a paper with this title.

#### DISCUSSION.

Dr. Phelps objected to the name, Non-Deforming Club-Foot, on the ground that all cases he had seen presented deformity.

Dr. Shaffer said that when he first called the attention of the profession to this subject, this name had suggested itself to him, because all the conditions of club-foot were present except the deformity which was so slight that it had hitherto escaped observation.

Dr. V. P. Gibney said that the condition described some years ago under the name of metatarsalgia might be confounded with non-deforming club-foot. These patients usually complain of pain after sitting for some time, as in the theatre. He had treated a few cases of non-deforming club-foot by division of the tendon and plantar fascia, over-correction, and retention in this position by plaster-of-Paris for a period of several weeks, and he had not been obliged to resort to extension subsequently. So far as he knew, these cases had not relapsed.

Dr. Shaffer said that in a series of twenty-two cases of metatarsalgia, he had relieved over one-half by the antero-posterior traction shoe alone. The inability to flex the ankle-joint brought the maximum pressure at the point of irritation, and hence, by producing a certain amount of forcible section at the ankle-joint, the pressure was brought upon other parts, thus removing the constant irritation which he thought was the chief etiological factor. He had permanently and completely relieved marked cases of non-deforming club-foot within one week by three or four applications of his traction apparatus. In some cases, deformity can be reduced at one sitting, but the muscles re-contract slightly, necessitating a more prolonged treatment. He did not believe that tenotomy was necessary in any case of non-deforming club-foot.

Dr. R. S. Sayre had found many cases of metatarsalgia to be dependent upon irritation of the pelvic nerves, and such cases had been relieved by galvanism with one pole over the sacrum, and the other over the ovarian region. In the treatment of non-deforming club-foot, he sometimes employed stretching, and sometimes tenotomy, depending upon the nature of the case. If the tendo Achillis or plantar fascia gave a reflex spasm when stretched to its utmost, while joint pressure was applied, his experience had been that tenotomy was necessary. If no reflex spasm was produced, the contracted tissues could be stretched.

Dr. Ridlon asked if the author considered the woman exhibited by Dr. Whitman, to be a pure non-deforming club-foot, and whether he would expect to fully relieve the disability and restore the full flexion by the use of a stretching apparatus.

Dr. Halsted Myers reported a case of non-deforming club-foot in a man, thirty-eight years of age, in which the etiology was unknown. The symptoms were unusual, in that although the ankle flexion was only stopped at 95°, the principal complaint was that the knee could not be fully extended. The patient also felt that he could not fully extend his thigh without much more effort than on the other side, and also felt that his pelvis was tilted up posteriorly. The man knew nothing of anatomy, yet these symptoms were reasonable theoretically, for shortened gastrocnemii might cause knee flexion, and this, in turn, thigh flexion, and this again, tilting of the pelvis up posteriorly. The shortening of the gastrocnemii was the only deformity apparent, yet the subjective symptoms were so annoying that the patient himself proposed forcible extension and fixation in bed, for months even, if necessary.

Dr. H. W. Berg said that he had seen many of the cases on which Dr. Shaffer's first paper was based, and not a little credit was due to him for having distinguished these cases from those of chronic rheumatism. Most of those described in the paper of the evening have been so quickly relieved, that he was inclined to think they were not congenital, for, after such a condition had lasted for many years, it did not seem reasonable that they should be relieved by one or two stretchings.

Dr. Hubbard, in closing the discussion, said that he had never seen a case of non-deforming club-foot which he thought would consent to an operation for the relief of the difficulty, as the patients did not ordinarily consider it of much importance. Nor could he recall a single case which had not been materially relieved after one or two stretchings, except in those which were rheumatic.

In answer to Dr. Ridlon, he would say that he considered the case presented by Dr. Whitman, a typical one of non-deforming club-foot of a rather pronounced type, but he had seen as bad, and even worse cases, relieved by persistent stretching. The treatment was prolonged in some instances by the re-contraction of the muscles, but just as india rubber yields after a certain number of stretchings, so these cases will be permanently relieved after the continued use of the traction shoe. In the case referred to in the paper, the condition had lasted for a long time, and the muscle was well developed, and the time of treatment was still further prolonged by the patient's intolerance of the stretching.

### A NEW APPARATUS FOR OVERCOMING THE ABDUCTION OF THE THIGH IN HIP-JOINT DISEASE.

Dr. Newton M. Shaffer exhibited a new apparatus which he had devised for the purpose of overcoming the abduction of the thigh in hip-joint disease, and at the same time avoiding the infliction of any traumatism upon the joint. It consisted of a thoracic attachment to the ordinary long hip splint, with an arrangement of curved levers actuated by a key, by which motion is imparted to the limb in a direction downwards and inwards, instead of as in other instruments of this class, inwards and upwards. This is the chief feature, and it is on this account that traumatism is avoided. It can be attached to any ordinary long traction splint, and like the thoracic part, it is to be used only as a temporary arrangement for reducing the deformity.

Dr. Phelps said that he was glad to see that Dr. Shaffer had come to recognize the fact that we cannot act upon the hip-joint with any degree of precision without taking hold of the thorax ; but he failed to see any necessity for such an apparatus in our armamentarium, as his lateral traction splint did the same thing, and no case of hip-joint disease need recover with angular deformity. Since he had devised and made use of his lateral traction fixation splint, which acts on the same principle as the apparatus just exhibited, he had not seen a case in his practice of angular deformity. If such a thoracic splint be applied after the deformity has once been overcome, recovery must take place without angular deformity.

Dr. Shaffer explained that the apparatus he had just pre sented was intended only as a temporary apparatus for overcoming persistent abduction of the thigh, and he considered it a very serious mistake to use the thoracic attachment in the ordinary treatment of hip-joint disease, because it limited the motion of the spinal column, and this would necessarily increase the strain upon the diseased joint. It was for this reason that he had discarded the thoracic addition to the hip splint many years ago. The idea of his new apparatus is to provide a temporary means of overcoming abduction, and it is only to be worn long enough to accomplish this purpose, and then it is so arranged that the abduction and thoracic portions can be readily removed, leaving the ordinary hip-splint, which permitted a free movement of the dorso-lumbar spine, and thus diminished the traumatism at the hip, which is best shown when a patient with hip-joint disease and dorso-lumbar caries attempts locomotion.

